# Normal reference values of three-dimensional speckle-tracking echocardiography-derived left atrial strain parameters (results from the MAGYAR-Healthy Study)

**DOI:** 10.1007/s10554-019-01559-z

**Published:** 2019-03-19

**Authors:** Attila Nemes, Árpád Kormányos, Péter Domsik, Anita Kalapos, Csaba Lengyel, Tamás Forster

**Affiliations:** 1 2nd Department of Medicine and Cardiology Center, Medical Faculty, Albert Szent-Györgyi Clinical Center, University of Szeged, Semmelweis Street 8, P.O. Box 427, 6725 Szeged, Hungary; 2 1st Department of Medicine, Medical Faculty, Albert Szent-Györgyi Clinical Center, University of Szeged, Szeged, Hungary

**Keywords:** Three-dimensional, Speckle-tracking, Echocardiography, Left atrium, Strain, Healthy

## Abstract

Left atrial (LA) size and function have been demonstrated to be important imaging biomarkers with powerful potential in predicting clinical outcome in several disorders. The angle-independent three-dimensional (3D) speckle-tracking echocardiography (3DSTE) has a capability for quantitative assessment of LA volumes and strains in 3D space at the same time from the same 3D acquired datasets. Therefore, the objective of the present study was to define normal values of 3DSTE-derived LA strains in healthy subjects. It was also examined whether there is any age- and gender-dependency of these parameters. The present study comprised 309 healthy volunteers, from which 87 were excluded due to inadequate image quality. The remaining group consisted of 222 subjects (mean age: 36.3 ± 13.7 years, 112 males). Complete two-dimensional echocardiography and 3DSTE have been performed in all cases. Peak circumferential strain (CS) increased with age with a decline > 50 years in females, in males CS remained almost unchanged. While peak longitudinal strain (LS) increased with age with unchanged parameters > 50 years, parallel increase in peak area strain (AS) with age could be demonstrated in both genders with a decline in females > 50 years. While CS and AS at atrial contraction increased with age in females, parallel decrease could be demonstrated in males. LS at atrial contraction increased with age especially in females. Normal values of 3DSTE-derived LA peak strains and strains at atrial contraction are demonstrated together with their age- and gender-dependency.

## Introduction

Left atrial (LA) size and function have been demonstrated to be important imaging biomarkers with powerful potential in predicting clinical outcome in several disorders [1]. Tissue Doppler imaging (TDI) and two-dimensional (2D) speckle-tracking echocardiography (STE) were found to be feasible and reproducible to evaluate LA mechanics by strain and strain rate analysis [2]. However, both techniques have technical limitations. The most important for TDI is its angle-dependency. Although LA wall has a 3D architecture and a complex motion during the cardiac cycle, echocardiographic speckles move out from the 2D plane during their tracking leading to difficulties in the detection of the real myocardial motion during 2DSTE [3]. The angle-independent three-dimensional (3D) STE solves these limitations due to its capability for quantitative assessment of LA volumes and strains in 3D space at the same time from the same 3D acquired datasets respecting LA phasic function [4].

Significant deterioration of LA strains has been demonstrated in several disorders [5–8]. Normal values of 3DSTE-derived LA strains, however, have never been assessed. Therefore, the objective of the present study was to define normal reference values of global and regional LA strains using 3DSTE in healthy subjects. It was also examined whether there is any age- and gender-dependency of these parameters.

## Patients and methods

### Patient population

The present study comprised 309 healthy volunteers, from which 87 were excluded due to inadequate image quality. The remaining group consisted of 222 subjects (mean age: 36.3 ± 13.7 years, 112 males). Normal subjects were medical students, hospital employees, and their relatives who participated on a voluntary basis. The eligibility criteria for healthy subjects included (1) no history of hypertension and normal blood pressure at the time of the echocardiographic examination; (2) no history of diabetes mellitus, hyperlipidemia, or cardiovascular disease; (3) no history of any medication use; (4) normal 2D echocardiographic results without valvular stenosis or grade > 1 regurgitation. All subjects were taken from the MAGYAR-Healthy Study (Motion Analysis of the heart and Great vessels bY three-dimensional speckle-tRacking echocardiography in Healthy subjects), which was created to assess normal values of 3DSTE-derived parameters among others (‘magyar’ means ‘Hungarian’ in Hungarian language). All subjects gave informed consent, the study complied with the Declaration of Helsinki and was approved by the institutional human research committee.

### Two-dimensional Doppler echocardiography

All healthy subjects underwent a complete transthoracic two-dimensional (2D) Doppler echocardiography using a Toshiba Artida™ echocardiography equipment (Toshiba Medical Systems, Tokyo, Japan) with a PST-30SBP (1–5 MHz) phased-array transducer. LA and left ventricular dimensions and ejection fraction were assessed according to recent guidelines [9]. Pulsed Doppler echocardiography was used for measurement of early (E) and late (A) diastolic transmitral flow velocities, while colour Doppler echocardiography was applied to exclude significant stenoses and regurgitations.

### Three-dimensional speckle-tracking echocardiography

**Fig. 1:**
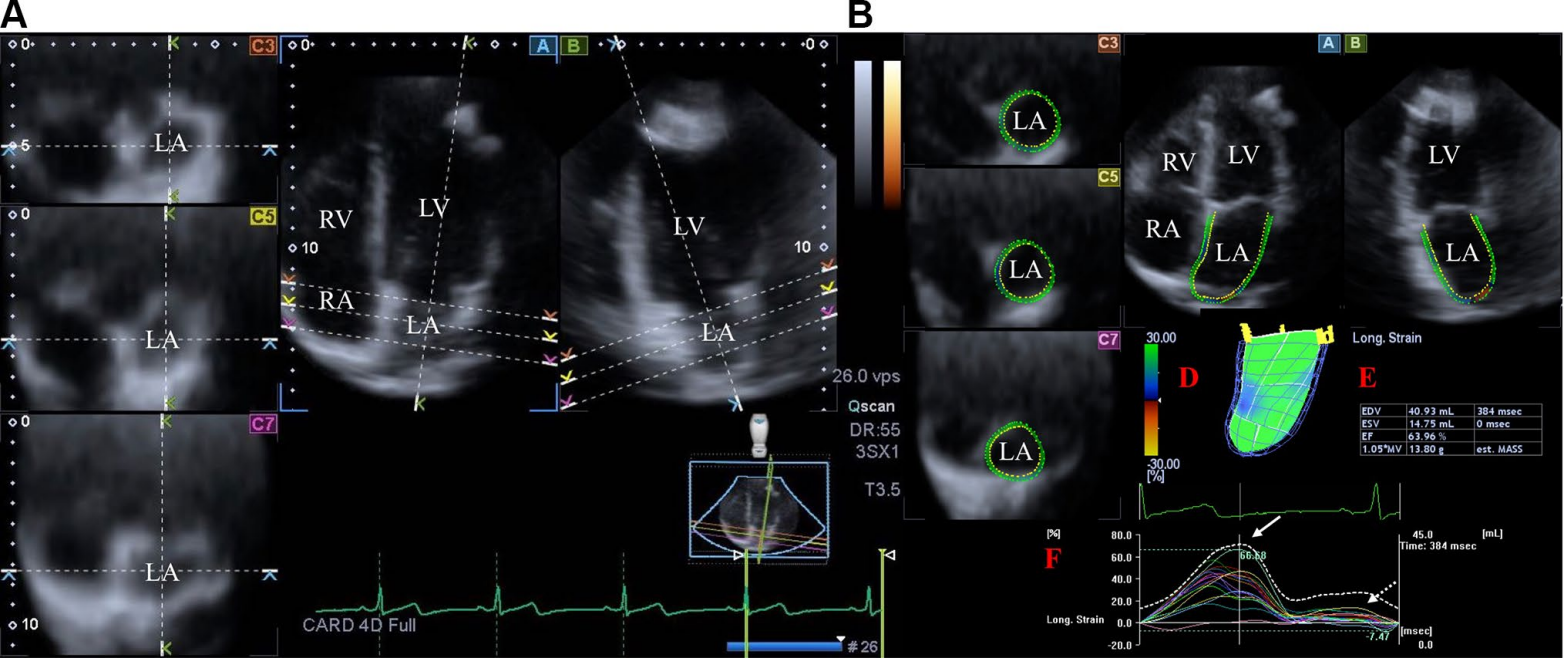
Images from three-dimensional (3D) full-volume dataset showing the left atrium (LA) in a healthy subject are demonstrated (**a, b**): (*A*) apical four-chamber view, (*B*) apical two-chamber view, (*C3*) short-axis view at basal, (*C5*) mid- and (*C7*) superior left atrial level. On **a**, dashed lines represent different planes optimalized on the long-axis and cross-section of the LA. On **b**, the semi-automated LA border definition, 3D “wire” reconstruction of the LA based on 3D speckle tracking echocardiographic analysis (red *D*) and LA volumetric data (red *E*) are presented. Coloured lines represent segmental LA strains while dashed white line represents LA volume changes over the cardiac cycle (red *F*). White arrow represents peak LA strain, while dashed arrow represents LA strain at atrial contraction. *LA* left atrium, *LV* left ventricle, *RA* right atrium, *RV* right ventricle

Toshiba Artida™ echocardiography equipment was used for 3DSTE-derived quantifications (Toshiba Medical Systems, Tokyo, Japan) with a PST-25SX matrix-array transducer with 3DSTE capability. Full-volume 3D datasets were created following acquisition of R-wave triggered wedge-shaped subvolumes from apical window during 6 cardiac cycles with constant RR interval. To improve spatial resolution, sector widths of the subvolumes were chosen to be as narrow as possible. During image acquisitions separate datasets for each chamber including the LA were created for later evaluations. Off-line analyses were performed for the LA using these LA-focused datasets. Acquired 3D datasets were analysed offline using 3D Wall Motion Tracking software version 2.7 (Toshiba Medical Systems, Tokyo, Japan). Apical four-chamber (AP4CH), two-chamber (AP2CH) and 3 short-axis LA views were chosen automatically by the software at end-diastole. Following optimisations, markers were set by the reader to the edges of the mitral annulus (MA) and the endocardial side of the apex (Fig. 1). Then the software automatically reconstructed the endocardial surface of the LA and a 3D cast of the LA was created for volumetric and strain analyses [5–8].

End-systolic maximum LA volume (V_max_, before mitral valve opening), early diastolic LA volume before atrial contraction (V_PreA_, at the time of the P-wave on the ECG) and end-diastolic minimum LA volume (V_min_, before mitral valve closure) were calculated for each subject. Using the same 3D datasets, several global, mean segmental and regional unidirectional [radial (RS), longitudinal (LS) and circumferential (CS)] and two complex/multidirectional [3D (3DS) and area (AS)] LA strains were obtained at the same time. Peak strains were measured during the LA reservoir phase in end-systole, while strains at atrial contraction were obtained at end-diastole (LA systole) during LA booster pump function (Fig. 1) [5–8].

### Statistical analysis

All data are reported as mean ± standard deviation or number and percentages. All values were considered significantly different at p < 0.05. Student’s *t* test was used for comparisons. Statistical analysis was performed by using RStudio (RStudio Team, RStudio: Integrated Development for R. RStudio, Inc., Boston, MA, 2015). For offline data analysis and graph creation, a commercial software package was used (MATLAB 8.6, The MathWorks Inc., Natick, MA, 2015).

## Results

### Demographic and two-dimensional echocardiographic data

A total of 222 healthy volunteers were included in the study in the following groups of subjects: 18–29 years (n = 99; mean age: 24.5 ± 2.6, 48 males), 30–39 years (n = 46; mean age: 34.0 ± 2.7 years, 16 males), 40–49 years (n = 26; mean age: 43.7 ± 4.4 years, 12 males) and > 50 years (n = 51, mean age: 57.3 ± 6.3 years, 18 males). Demographic and 2D echocardiographic data were in normal ranges as demonstrated in Table 1.

**Table 1:** Clinical, two-dimensional and volumetric three-dimensional speckle-tracking echocardiographic data of healthy subjects

	Data
n	222
Age (years)	36.3 ± 13.7
Male gender (%)	112 (50)
Weight (kg)	72.9 ± 17.5
Height (m)	172.7 ± 10.9
Body surface area (kg/m^2^)	1.86 ± 0.23
Two-dimensional echocardiography	
Left atrium (mm)	37.9 ± 3.8
Left ventricular end-diastolic diameter (mm)	48.3 ± 3.7
Left ventricular end-diastolic volume (ml)	107.1 ± 26.5
Left ventricular end-systolic diameter (mm)	32.5 ± 3.6
Left ventricular end-systolic volume (ml)	38.3 ± 10.5
Interventricular septum (mm)	9.2 ± 1.2
Left ventricular posterior wall (mm)	9.4 ± 1.6
E (cm/s)	77.9 ± 16.4
A (cm/s)	62.2 ± 15.3
Left ventricular ejection fraction (%)	64.9 ± 3.9
Three-dimensional speckle-tracking echocardiography	
Maximum left atrial volume (ml)	41.4 ± 13.4
Preatrial contraction left atrial volume (ml)	28.5 ± 12.3
Minimum left atrial volume (ml)	19.9 ± 8.6

### 3DSTE-derived peak LA strains (reservoir function)

**Fig. 2:**
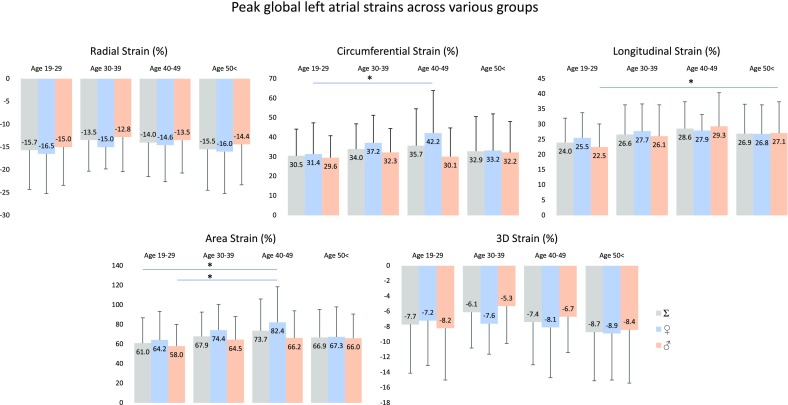
Global peak left atrial strains in males and females over decades. Lines with a star represent significant difference between strains

Peak global LA strains and their gender dependency over decades are demonstrated in Figs. 2 and 4. No significant differences could be demonstrated in strains between the genders, only tendencies could be seen. RS declined with age in females, while an increase in RS could be demonstrated in males > 40 years. CS increased with age with a decline > 50 years in females, in males CS remained almost unchanged. While LS increased with age with unchanged parameters > 50 years, parallel increase in AS with age could be demonstrated in both genders with a decline in females > 50 years. 3DS did not change over the years in females, but an early reduction in males with a later increase could be seen. Peak mean segmental LA strains and differences in regional peak strains over decades are demonstrated in Tables 2 and 3.

**Fig. 3:**
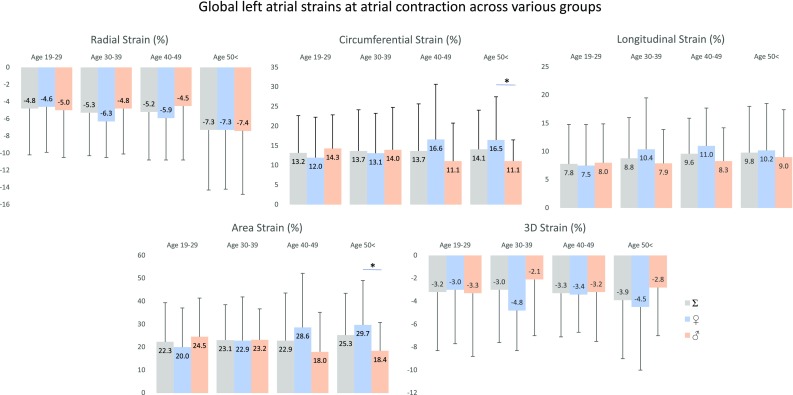
Global left atrial strains at atrial contraction in males and females over decades. Lines with a star represent significant difference between strains

**Table 2:** Age-dependency of three-dimensional speckle-tracking echocardiography-derived peak mean segmental left atrial strain parameters and mean segmental left atrial strain parameters at atrial contraction

	All	Aged 19–29 years	Aged 30–39 years	Aged 40–49 years	Aged > 50 years
Peak mean segmental strains					
RS (%)	− 19.5 ± 7.0	− 20.4 ± 7.4	− 18.4 ± 5.9	− 19.0 ± 6.2	− 18.9 ± 7.1
CS (%)	36.7 ± 14.3	35.6 ± 12.9	37.8 ± 12.2	40.0 ± 17.2^†^	36.4 ± 16.7
LS (%)	29.2 ± 8.3	28.1 ± 7.3	30.0 ± 9.1	31.3 ± 7.9	29.3 ± 9.2
3DS (%)	− 12.8 ± 5.2	− 13.3 ± 5.4	− 11.8 ± 4.9	− 12.4 ± 5.0	− 12.8 ± 5.4
AS (%)	71.7 ± 26.3	68.9 ± 25.1	73.9 ± 24.4	79.1 ± 30.2^‡^	71.5 ± 27.7
Mean segmental strains at atrial contraction					
RS (%)	− 8.2 ± 4.9	− 7.6 ± 4.7	− 7.8 ± 4.2	− 8.5 ± 5.7	− 9.3 ± 5.3
CS (%)	15.0 ± 8.4	14.0 ± 7.2	16.0 ± 8.3	15.0 ± 11.7	16.0 ± 8.7
LS (%)	9.9 ± 5.2	9.2 ± 5.1	9.9 ± 5.3	10.6 ± 4.1	10.9 ± 5.4
3DS (%)	− 5.4 ± 4.3	− 5.0 ± 4.1	− 5.1 ± 4.1	− 5.6 ± 4.3	− 6.2 ± 4.8
AS (%)	25.8 ± 14.4	24.0 ± 13.2	26.5 ± 13.6	27.2 ± 16.4	28.0 ± 16.0

^†^p < 0.05 vs. peak CS Aged 19–29 years

^‡^p < 0.05 vs. peak AS Aged 19–29 years

**Table 3:** Age-dependency of three-dimensional speckle-tracking echocardiography-derived peak regional left atrial strain parameters

	All	Aged 19–29 years	Aged 30–39 years	Aged 40–49 years	Aged > 50 years
RS_basal_ (%)	− 18.2 ± 8.9	− 19.3 ± 9.5	− 16.5 ± 7.5	− 16.2 ± 7.2**	− 18.5 ± 9.6
RS_midatrial_ (%)	− 19.3 ± 7.7*	− 20.3 ± 8.5	− 17.8 ± 6.2	− 18.2 ± 7.1**	− 19.3 ± 7.4
RS_superior_ (%)	− 21.2 ± 12.0*	− 22.2 ± 12.4	− 19.8 ± 12.2	− 24.3 ± 12.6	− 19.3 ± 10.6
CS_basal_ (%)	40.5 ± 14.8^††^	39.2 ± 12.1^†^	41.7 ± 14.9^†††^	42.1 ± 15.9	41.2 ± 18.5^††††^
CS_midatrial_ (%)	31.5 ± 12.8	29.7 ± 11.7	32.7 ± 11.2	34.9 ± 13.3†	32.2 ± 15.3
CS_superior_ (%)	38.8 ± 26.1^††^	39.2 ± 25.6^†^	38.3 ± 22.1	44.4 ± 37.1	35.8 ± 23.6
LS_basal_ (%)	22.1 ± 11.2^‡‡^	20.3 ± 10.3	22.8 ± 11.6^‡‡‡‡^	21.6 ± 8.5^‡‡‡‡‡/‡‡‡‡‡‡^	25.0 ± 12.9^‡/‡‡‡‡‡‡‡^
LS_midatrial_ (%)	36.9 ± 13.5	35.5 ± 11.6^‡^	38.4 ± 14.1	41.0 ± 17.8	36.4 ± 13.7
LS_superior_ (%)	27.0 ± 15.1^‡‡^	26.7 ± 14.2^‡‡‡^	26.2 ± 14.9^‡‡‡‡^	31.3 ± 17.2^‡‡‡‡‡^	26.3 ± 15.8^‡‡‡‡‡‡‡^
3DS_basal_ (%)	− 12.8 ± 7.2	− 13.6 ± 7.7	− 10.9 ± 5.7	− 10.9 ± 5.0^§^	− 13.6 ± 10.2
3DS_midatrial_ (%)	− 11.8 ± 5.8	− 12.3 ± 6.3	− 10.4 ± 14.9	− 11.3 ± 5.8^§^	− 12.3 ± 5.4
3DS _superior_ (%)	− 13.8 ± 9.0	− 14.0 ± 8.9	− 12.8 ± 9.8	− 15.8 ± 9.4	− 13.2 ± 8.4
AS_basal_ (%)	61.6 ± 24.1	57.7 ± 19.8	63.3 ± 24.8	61.8 ± 23.2	67.5 ± 29.6^$$^
AS_midatrial_ (%)	74.0 ± 27.0	69.9 ± 25.1^$$^	78.3 ± 30.4^$$$$^	82.1 ± 25.4^$$$^	74.5 ± 26.9
AS_superior_ (%)	82.2 ± 62.6^$^	82.4 ± 59.2^$$/$$$^	79.5 ± 54.9	100.7 ± 94.8	75.4 ± 54.1

*p < 0.05 vs. RS_basal_ All; ** p < 0.05 vs. RS_superior_ Aged 40–49 years

^†^p < 0.05 vs. CS_midatrial_ Aged 19–29 years; ^††^p < 0.05 vs. CS_midatrial_ All; ^†††^p < 0.05 vs. CS_midatrial_ Aged 30–39 years; ^††††^ p < 0.05 vs. CS_midatrial_ Aged 50 < years

^‡^p < 0.05 vs. LS_basal_ Aged 19–29 years; ‡‡ p < 0.05 vs. LS _midatrial_ All_;_^‡‡‡^p < 0.05 vs. LS _midatrial_ Aged 19–29 years; ^‡‡‡‡^ p < 0.05 vs. LS _midatrial_ Aged 30–39 years; ^‡‡‡‡‡^p < 0.05 vs. LS_midatrial_ Aged 40–49 years; ^‡‡‡‡‡‡^p < 0.05 vs. LS_superior_ Aged 40–49 years; ^‡‡‡‡‡‡‡^p < 0.05 vs. LS_midatrial_ Aged 50 < years

^§^p < 0.05 vs. 3DS_superior_ Aged 40–49 years

^$^p < 0.05 vs. AS_basal_ All; $$ p < 0.05 vs. AS_basal_ Aged 20–29; ^$$$^p < 0.05 vs. AS _midatrial_ Aged 20–29; ^$$$$^p < 0.05 vs. AS _basal_ Aged 30–39

### 3DSTE-derived LA strains at atrial contraction

**Fig. 4:**
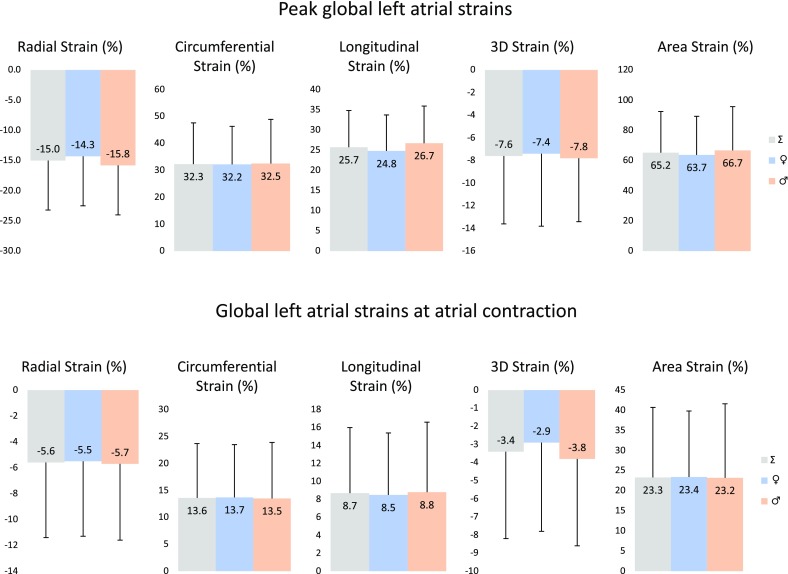
Gender dependency of global peak left atrial strains and left atrial strains at atrial contraction over decades

Global LA strains at atrial contraction and their gender dependency over decades are demonstrated in Figs. 3 and 4. No significant changes in any of strains could be demonstrated between different age groups, only tendentious alterations could be confirmed. Higher RS at atrial contraction could be seen in older ages. While CS and AS at atrial contraction increased with age in females, parallel decrease could be demonstrated in males. LS at atrial contraction increased with age especially in females. 3DS at atrial contraction proved to be highest in females in age group 30–39 years. Mean segmental LA strains at atrial contraction and differences in regional peak strains over decades are demonstrated in Tables 3 and 4.

**Table 4:** Age-dependency of three-dimensional speckle-tracking echocardiography-derived regional left atrial strain parameters at atrial contraction

	All	Aged 19–29 years	Aged 30–39 years	Aged 40–49 years	Aged > 50 years
RS_basal_ (%)	− 7.9 ± 5.8	− 7.4 ± 5.6	− 7.4 ± 5.5	− 6.9 ± 5.0	− 9.7 ± 6.6
RS_midatrial_ (%)	− 7.9 ± 5.4	− 7.5 ± 5.1	− 7.5 ± 5.0	− 8.5 ± 6.9	− 8.8 ± 5.4
RS_superior_ (%)	− 9.1 ± 8.4	− 8.4 ± 8.6	− 8.9 ± 8.4	− 10.8 ± 9.2	− 9.6 ± 7.6
CS_basal_ (%)	16.5 ± 9.0^††^	15.1 ± 7.9^†††^	18.7 ± 9.9^†/††††^	17.0 ± 11.0	16.7 ± 8.8
CS_midatrial_ (%)	13.0 ± 8.4	11.9 ± 7.5	12.8 ± 7.5	13.2 ± 10.3	14.8 ± 9.5
CS_superior_ (%)	15.9 ± 14.6^††^	15.7 ± 13.1^†††^	15.5 ± 15.3	14.8 ± 20.3	16.9 ± 13.6
LS_basal_ (%)	7.5 ± 5.2	6.8 ± 5.1	7.9 ± 5.6	7.9 ± 4.6	8.5 ± 5.0^‡^
LS_midatrial_ (%)	11.4 ± 7.5^‡‡^	10.2 ± 7.1^‡^	11.9 ± 7.9^‡‡‡^	12.6 ± 6.8^‡‡‡‡^	12.6 ± 8.0^‡‡‡‡‡^
LS_superior_ (%)	11.0 ± 8.7^‡‡^	10.7 ± 8.4^‡^	10.0 ± 9.0	11.5 ± 9.5	12.1 ± 8.5^‡‡‡‡‡^
3DS_basal_ (%)	− 5.4 ± 5.4	− 5.0 ± 5.2	− 4.8 ± 5.1	− 4.5 ± 4.6^§^	− 6.8 ± 6.4
3DS_midatrial_ (%)	− 5.0 ± 4.7	− 4.7 ± 4.8	− 4.8 ± 4.4	− 4.9 ± 5.5^§^	− 5.6 ± 4.3
3DS_superior_ (%)	− 6.0 ± 7.3	− 5.4 ± 6.8	− 5.9 ± 8.0	− 8.5 ± 7.5	− 6.2 ± 7.4
AS_basal_ (%)	22.7 ± 13.0	20.3 ± 10.9^$$$$$^	24.6 ± 14.5	27.1 ± 14.1^$^	23.9 ± 14.1
AS_midatrial_ (%)	25.8 ± 15.3^$$$^	23.3 ± 15.3^$$$$$^	26.5 ± 13.3	27.0 ± 14.0	29.2 ± 16.9^$$^
AS_superior_ (%)	30.6 ± 29.1^$$$/$$$$^	30.4 ± 26.8	29.6 ± 31.6	27.8 ± 35.8	32.5 ± 27.8

^†^p < 0.05 vs. CS _basal_ Aged 19–29 years; ^††^p < 0.05 vs. CS _midatrial_ All; ^†††^p < 0.05 vs. CS _midatrial_ Aged 19–29; ^††††^p < 0.05 vs. CS _midatrial_ Aged 30–39

^‡^p < 0.05 vs. LS_basal_ Aged 19–29 years; ^‡‡^p < 0.05 vs. LS_basal_ All; ^‡‡‡^p < 0.05 vs. LS_basal_ Aged 30–39 years; ^‡‡‡‡^p < 0.05 vs. LS_basal_ Aged 40–49 years; ^‡‡‡‡‡^p < 0.05 vs. LS_basal_ Aged 50 < years

^§^p < 0.05 vs. 3DS_superior_ Aged 40–49 years

^$^p < 0.05 vs. AS_basal_ Aged 20–29; ^$$^p < 0.05 vs. AS_midatrial_ Aged 20–29; ^$$$^p < 0.05 vs. AS_basal_ All; ^$$$$^p < 0.05 vs. AS_midatrial_ All; ^$$$$$^p < 0.05 vs. AS_superior_ Aged 19–29

### Feasibility of 3DSTE-derived LA measurements

3DSTE measurements were carried out between 2011 and 2016, during this period feasibility improved as the operators gained experience. During the whole 5-year period 222 measurements were successfully performed out of 309 (72%), from which 56 measurements proved to be successful in 66 subjects (85%) in the last year.

## Discussion

3DSTE is the most recent echocardiographic technique using ‘block-matching’ algorhythm for imaging [4, 10, 11]. The method permits of merging both benefits of 3D and STE imaging: enables to see heart chambers in 3D space by creating a virtual model of a particular chamber and by using this 3D cast, several uni- and multidirectional (dimensional) strains could be calculated over volumetric assessments at the same time [4, 10, 11]. Recently, normal reference values of global and regional LV strains using 3DSTE have already been demonstrated [12]. Due to absence of widely used LA echocardiographic segmentation, the same methodology applied for LV was used for strain assessment in the present study. The presented 3DSTE-derived LA strain analysis has already been validated against 2D echocardiography [13], 2DSTE [14] and volumetric real-time 3D echocardiography [15]. Recently, inter- and intraobserver agreements with correlation coefficients have already been demonstrated for 3DSTE-derived LA strain parameters [7].

LA has a distinct phasic function during the cardiac cycle: it works as a reservoir in systole, it acts as a conduit in early diastole and it works as a booster pump in late diastole helping LV filling through active contraction [16]. During 3DSTE, peak LA strain and LA strain at atrial contraction representing systolic reservoir and late-diastolic booster pump LA functions were calculated automatically from the two-peak curve created by the software using the 3D LA cast in each subject, respectively [16].

In recent studies, significant alterations in 3DSTE-derived LA strain parameters could be demonstrated in several disorders suggesting disease-specific pattern of LA dysfunction [5]. However, LA strains of healthy controls showed differences in these studies suggesting that selection of healthy volunteers and their clinical parameters could have an effect on comparisons [6–8]. Therefore, the current study aimed to establish normal reference ranges of different LA strains using 3DSTE. Over different global and mean segmental LA strains, basal, midatrial and superior (‘apical’) regional strains were defined in males and females among different age groups. In contrast with normal values of 3DSTE-derived LV strains, wide range for LA strains could be demonstrated (large standard deviation) [12]. It is likely due to the thin LA wall in combination with limited spatial resolution. Differences were found in LA strains at different LA levels suggesting functional non-uniformity similarly to LV as well [12].

In a recent meta-analysis, the following normal reference ranges for different STE-derived LA strains were revealed: for reservoir (peak) strain of 39% (95% CI 38–41%, from 40 articles), for conduit strain of 23% (95% CI 21–25%, from 14 articles), and for contractile strain of 17% (95% CI 16–19%, from 18 articles) [17]. LA strain was retrospectively measured by 3D echocardiography for measuring LA strains in healthy children as well using a commercial speckle-tracking package applied to the LA to compute global 3D principal (3DS), longitudinal (GLS), and circumferential (GCS) strains in a recent study [18]. In healthy children, all components of LA strain were found to decline modestly with age. To the best of the authors’ knowledge, this is the first time to demonstrate normal reference values of differed 3DSTE-derived LA strains demonstrating their age-dependency. It has also been demonstrated that LA strains have no obvious gender-dependency (except in older ages), but tendencies could be detected together with regional differences.

### Limitations

The present study did not aim to assess normal values of LA volumetric data and strain rate parameters. Moreover, only peak strains and strains at atrial contraction were compared. As mentioned before, inter- and intraobserver agreements with correlation coefficients for LA strains have already been confirmed. Spatial resolution of 3DSTE is relatively poor compared to that of 2D echocardiography. Although healthy subjects were examined relatively high number of them was excluded. Exclusion was mainly based on image quality. Narrow echocardiographic window (as the 3DSTE transducer is more sizeable than its 2D counterpart) and the operators’ inexperience (at the beginning of the study) could be considered as the most important reasons behind exclusion. It is also worthy to note in lesser part that body habitus was also a reason in some cases for exclusion, however extreme obesity was considered pathologic and these patients were not enrolled in the present study.

## Conclusion

Normal reference values of 3DSTE-derived LA peak strains and strains at atrial contraction are demonstrated together with their age- and gender-dependency.
